# Life expectancy of cancer patients in China

**DOI:** 10.1016/j.mmr.2026.100045

**Published:** 2026-06-15

**Authors:** Chang-Fa Xia, Jing Yang, Jin-Hui Zhou, Yong-Jie Xu, Wan-Qing Chen

**Affiliations:** a Office of Cancer Screening, National Cancer Center/National Clinical Research Center for Cancer/Cancer Hospital, Chinese Academy of Medical Sciences and Peking Union Medical College, Beijing 100021, China; b Department of Radiation Oncology, National Cancer Center/National Clinical Research Center for Cancer/Cancer Hospital, Chinese Academy of Medical Sciences and Peking Union Medical College, Beijing 100021, China; c National Central Cancer Registry, National Cancer Center/National Clinical Research Center for Cancer/Cancer Hospital, Chinese Academy of Medical Sciences and Peking Union Medical College, Beijing 100021, China

**Keywords:** Cancer, Life expectancy (LE), Years of life lost (YLL), Population-based, China

## Abstract

**Background:**

A cancer diagnosis is usually associated with a substantial loss of life years. However, few studies have quantified life expectancy (LE) and years of life lost (YLL) among cancer patients, particularly in China. This study aims to estimate LE and YLL by cancer type, sex, age at diagnosis, and attained age (i.e., the age a cancer survivor has reached at a given time) for cancer patients in China.

**Methods:**

This is a comparative assessment based on population-based cancer registration and death surveillance in China. Data on all-cause deaths, cancer cases, and relative survival in 2021 were obtained from publicly available reports released by the National Cancer Center and the Chinese Center for Disease Control and Prevention. Life tables for the general population were constructed using age-specific all-cause mortality rates, whereas relative survival rates and parameterized long-term excess hazard functions were used to construct life tables for cancer patients. We used the standard period life table method to estimate LE of both the general population and cancer patients, assuming that mortality probabilities remained constant over time. The YLL was the LE difference between cancer patients and the sex- and age-matched general population.

**Results:**

For cancer patients diagnosed at the median age of 64 years, the LE is 8.8 years [95% confidence interval (CI) 8.7−9.0], corresponding to a YLL of 9.2 years (95% CI 9.0−9.3). Male patients have an LE and YLL of 6.7 years (95% CI 6.6−6.8) and 9.8 years (95% CI 9.7−9.9), respectively, while females have 11.2 years (95% CI 11.1−11.4) and 8.4 years (95% CI 8.3−8.6). Thyroid cancer had the highest LE [31.9 years (95% CI 31.5−32.0)], while pancreatic cancer had the lowest [2.6 years (95% CI 2.4−2.8)]. Female patients had better LEs than males across 20 cancer types that affect both sexes. Younger patients experienced greater YLL, except in cases of thyroid cancer, oropharyngeal cancer, ovarian cancer, and male bladder cancer. LE gradually increased in patients who survived the first three years; thereafter, the decline depended on attained age, approaching that of the general population.

**Conclusions:**

LE of cancer patients in China varied by sex, cancer type, age at diagnosis, and attained age. Cancer patients, healthcare providers, and policymakers may incorporate these estimates into their decision-making.

## 1 Background

Life expectancy (LE) is a summary measure of mortality that estimates the average number of years a population is expected to live. It is not only widely used in demographic analysis but also serves as a compelling summary measure for public health [1]. Compared to the general population, cancer patients have a higher excess mortality risk and experience a substantial loss of life years. The LE indicator integrates the complete mortality profile of the observed population and is unaffected by the age structure of the population [2]. Therefore, comparing the differences in LE and healthy LE between cancer patients and the general population directly quantifies the true burden of cancer [3]. This approach provides real-world data on cancer’s actual impact on a specific population and reveals how a cancer diagnosis affects future life prospects.

The relative survival rate (i.e., the ratio of a cancer patient’s observed survival rate to that of the general population) is commonly used to assess the LEs of cancer patients [4]. In the early stages after diagnosis, when excess mortality risk is high, this metric provides valuable insight. However, as the time since a cancer diagnosis increases, the risk of cancer-related death declines and may become comparable to that of the general population [5], [6]. In such cases, the relative survival rate can exceed one [7]. Because it is a relative measure, the relative survival rate does not directly quantify the absolute burden of cancer [8]. In contrast, years of life lost (YLL) offers a more intuitive way for patients to understand the life-threatening impact of cancer [9].

The LE of cancer patients is associated with numerous factors. Within a given country or healthcare setting, LE of cancer patients is determined by both patient- and cancer-specific factors. Patient-specific factors include age at diagnosis, sex, health literacy, healthy behaviors, socioeconomic status, accessibility of cancer care, and the patient’s attained age (i.e., the age a cancer survivor has reached at a given time) [10], [11], [12]. Cancer-specific factors include cancer type, anatomical subsite, cancer stage, tumor grade, biomarkers, and histologic subtype [13], [14]. In addition to patient- and cancer-specific factors, LE for cancer also varies significantly between countries and regions, as evidenced by differences in cancer relative survival rates [15]. While many studies have reported LE estimates for selected cancer types in various countries, such as the United States, England, Italy, and Australia [5], [6], [10], [16], [17], [18], [19], estimates on LE and YLL among cancer patients in China remain unavailable [20], [21].

In China, the general population’s LE at birth has gradually increased to 78.2 years in 2021 [22]. Similarly, the 5-year relative survival rate for cancer patients has also increased, reaching 43.7% in 2019−2021 [23]. Despite these improvements in cancer survival, cancer remains a major contributor to the deficit in LE. This study aims to estimate the LE of patients across all cancer types, stratified by sex, age at diagnosis, and attained age, and to compare these estimates with those of the age- and sex-matched general population, thereby revealing the YLL among cancer patients.

## 2 Methods

### 2.1 Data sources

This study is a population-based comparative assessment conducted in China. Data on all-cause deaths, cancer incidence, and relative survival rates of cancer patients were obtained from publicly available reports (**Additional file 1:** Tables S1-S5) [22], [23], [24], [25], [26], [27], [28]. To align with the latest cancer survival data for 2021, we collected contemporaneous data released by the National Cancer Center and the Chinese Center for Disease Control and Prevention. Specifically, sex- and age-specific all-cause mortality rates for the general population in 2021 were collected from the China Health Statistical Yearbook [22] and the Chinese Cause of Death Surveillance dataset [24]. Population data were extracted from the China Population Census Yearbook [25], and the number of births in 2021 was collected from the China Statistical Yearbook [26]. Sex-, cancer type-, and age-specific cancer incidence data were retrieved from the GLOBOCAN 2022 database [27], except for bone and other thoracic organ cancers, which were sourced from the 2022 China Cancer Registry Annual Report [28]. Sex-, cancer type-, and age-specific relative survival rates for cancer patients from 2019−2021 were informed from 281 population-based cancer registries [23]. This study was approved by the Ethics Committee of National Cancer Center/Cancer Hospital, Chinese Academy of Medical Sciences, and Peking Union Medical College (22/489-3691).

### 2.2 Statistical analysis

We used the standard period life table method to estimate LE of both the general population and cancer patients [5], [17], [29]. LE remaining at a given age was calculated through a series of calculations based on age-specific mortality probabilities, organized in the rows and columns of a life table (**Additional file 1:** Table S6). The period life table was constructed from mortality rates observed in a single calendar year, and it assumed that individuals would be exposed to these mortality conditions throughout the remainder of their lives; consequently, this approach did not account for potential future changes in mortality. By contrast, cohort life tables were based on age-specific probabilities of death derived directly from the observed mortality experience of a defined cohort. Specifically, cohort life tables integrated the cohort’s historically observed mortality rates with projections of future mortality trends. Age-specific annual mortality risks for all birth cohorts alive in 2021 were used to construct a life table, from which the general population’s LE was calculated for each age. The life table was truncated at age 100 years (i.e., the population still alive at age 99 years was assumed to die upon reaching age 100 years). For cancer patients, LE was calculated in 4 steps [5], [17], [30]. Firstly, sex- and age-specific relative survival rates of cancer patients in 2019−2021 were used to parameterize long-term excess hazard functions [31]. Secondly, the excess hazard for cancer patients was then derived by sex, age at diagnosis, and attained age. Thirdly, cancer excess mortality was combined with general population mortality to estimate the overall all-cause mortality risk in cancer patients. Finally, the LE for cancer patients was then calculated using the same method applied to the general population. The YLL was calculated as the difference in LE between cancer patients and a sex- and age-matched general population.

The LE estimates were highly sensitive to the age at diagnosis of cancer patients. To calculate the average LE, we needed to predefine a representative age at diagnosis for each cancer type. Therefore, we calculated the LE at the median age of diagnosis for each cancer. For consistency, the same median age was applied to both males and females within a given cancer type to ensure comparability. The combined LE for males and females was calculated by weighting the male and female LE estimates according to the male-to-female ratio of cancer cases for that type [17].

Within specified sex and age strata, the LE and YLL estimates for each cancer type were ranked according to their corresponding quantile values. 95% confidence interval (CI) of LE estimates for cancer patients was computed by the delta method [5], accounting for the variation of relative survival rates. All statistical analyses were performed using R software (version 4.4.2, R project).

## 3 Results

### 3.1 LE and YLL at the median age of cancer diagnosis

In 2021, cancer patients in China had a LE of 8.8 years (95% CI 8.7−9.0) at the median age at diagnosis (i.e., 64 years). Compared to the general Chinese population, YLL of cancer patients was 9.2 years (95% CI 9.0−9.3). Diagnosed at a median age of 64 years, male patients had a lower LE than female patients [6.7 years (95% CI 6.6−6.8) vs*.* 11.2 years (95% CI 11.1−11.4)] but a greater YLL [9.8 years (95% CI 9.7−9.9) vs. 8.4 years (95% CI 8.3−8.6)]. The median age at diagnosis varied across cancer types, ranging from the highest in prostate cancer (74 years) to the lowest in testicular cancer (43 years). Among patients diagnosed at their specific median ages, thyroid cancer had the highest LE [31.9 years (95% CI 31.5−32.0) at a median diagnosis age of 48 years], while pancreatic cancer had the lowest [2.6 years (95% CI 2.4−2.8) at a median diagnosis age of 69 years]. Female patients had better LEs than males across 20 cancer types that affect both sexes. However, the YLL was not necessarily higher than that of female patients (Table 1).

**Table 1:** Life expectancy (LE) and years of life lost (YLL) among cancer patients at the median age in China.

**Cancer site**	**ICD-10**	**Median age (year)**	**Both**		**Male**		**Female**	
			**LE (year, 95% CI)**	**YLL (year, 95% CI)**	**LE (year, 95% CI)**	**YLL (year, 95% CI)**	**LE (year, 95% CI)**	**YLL (year, 95% CI)**
Oral/Pharynx	C00−10, 12−14	64	7.9 (7.5−8.3)	9.5 (9.1−9.9)	6.4 (6.1−6.7)	10.1 (9.8−10.4)	11.5 (10.9−12.1)	8.2 (7.6−8.8)
Nasopharynx	C11	55	12.7 (12.0−13.4)	12.1 (11.4−12.8)	11.3 (10.8−11.9)	12.3 (11.7−12.9)	16.1 (15.1−17.1)	11.6 (10.6−12.6)
Esophagus	C15	69	4.7 (4.5−4.9)	8.9 (8.7−9.0)	4.2 (4.1−4.4)	8.7 (8.5−8.8)	6.2 (5.9−6.4)	9.4 (9.1−9.6)
Stomach	C16	68	5.9 (5.8−6.1)	8.5 (8.3−8.7)	5.4 (5.3−5.6)	8.1 (8.0−8.3)	7.0 (6.8−7.3)	9.3 (9.1−9.5)
Colorectum	C18−21	67	8.7 (8.5−8.9)	6.8 (6.6−6.9)	7.7 (7.5−7.8)	6.6 (6.4−6.8)	10.2 (9.9−10.4)	7.0 (6.7−7.2)
Liver	C22	64	4.4 (4.2−4.5)	13.0 (12.8−13.2)	4.1 (3.9−4.3)	12.4 (12.2−12.5)	5.0 (4.8−5.3)	14.6 (14.4−14.9)
Gallbladder	C23−24	70	3.8 (3.5−4.0)	10.0 (9.7−10.2)	3.3 (3.1−3.5)	8.9 (8.8−9.1)	4.1 (3.8−4.3)	10.7 (10.4−10.9)
Pancreas	C25	69	2.6 (2.4−2.8)	11.4 (11.2−11.6)	2.5 (2.3−2.6)	10.4 (10.3−10.6)	2.8 (2.6−3.0)	12.7 (12.5−12.9)
Larynx	C32	65	7.7 (7.3−8.1)	8.3 (7.9−8.7)	7.5 (7.2−7.9)	8.2 (7.8−8.5)	9.3 (8.4−10.2)	9.6 (8.6−10.5)
Lung	C33−34	67	5.2 (5.0−5.3)	10.2 (10.1−10.3)	3.9 (3.8−4.1)	10.3 (10.2−10.5)	7.2 (7.0−7.4)	10.0 (9.8−10.2)
Other thoracic organs	C37−38	61	9.4 (8.7−10.1)	10.8 (10.2−11.5)	7.5 (6.9−8.0)	11.3 (10.8−11.8)	12.3 (11.4−13.2)	10.0 (9.2−10.9)
Bone	C40−41	64	5.7 (5.2−6.2)	12.2 (11.7−12.6)	4.7 (4.2−5.1)	11.8 (11.4−12.2)	7.1 (6.5−7.7)	12.6 (12.0−13.2)
Melanoma of skin	C43	66	7.4 (6.8−8.0)	9.1 (8.5−9.7)	6.0 (5.5−6.5)	9.0 (8.5−9.5)	8.8 (8.1−9.5)	9.1 (8.5−9.8)
Breast	C50	54	22.1 (21.6−22.6)	6.5 (6.0−7.0)	14.8 (13.3−16.1)	9.7 (8.4−11.2)	22.2 (21.7−22.7)	6.5 (5.9−7.0)
Cervix	C53	54	20.1 (19.5−20.6)	8.6 (8.0−9.1)	—	—	20.1 (19.5−20.6)	8.6 (8.0−9.1)
Uterus	C54−55	55	21.7 (21.1−22.3)	6.0 (5.5−6.6)	—	—	21.7 (21.1−22.3)	6.0 (5.5−6.6)
Ovary	C56	56	13.4 (12.8−14.0)	13.4 (12.8−14.0)	—	—	13.4 (12.8−14.0)	13.4 (12.8−14.0)
Prostate	C61	74	5.5 (5.4−5.6)	4.2 (4.1−4.4)	5.5 (5.4−5.6)	4.2 (4.1−4.4)	—	—
Testis	C62	43	26.8 (25.1−28.5)	7.5 (5.7−9.2)	26.8 (25.1−28.5)	7.5 (5.7−9.2)	—	—
Kidney	C64−66, 68	62	12.4 (11.9−12.8)	6.9 (6.4−7.3)	11.1 (10.7−11.4)	7.0 (6.6−7.3)	14.7 (14.2−15.3)	6.7 (6.2−7.3)
Bladder	C67	70	7.7 (7.5−7.9)	5.1 (4.8−5.3)	7.3 (7.1−7.5)	4.9 (4.7−5.1)	9.2 (8.8−9.6)	5.6 (5.2−5.9)
Brain	C70−72	60	8.1 (7.6−8.5)	13.4 (12.9−13.8)	5.7 (5.4−6.1)	13.9 (13.5−14.2)	10.3 (9.8−10.8)	12.9 (12.4−13.4)
Thyroid	C73	48	31.9 (31.5−32.0)	1.1 (1.1−1.6)	28.1 (27.6−28.2)	1.6 (1.5−2.2)	33.3 (32.9−33.4)	1.0 (0.9−1.4)
Lymphoma	C81−85, 88, 90, 96	65	6.7 (6.4−7.0)	10.4 (10.1−10.6)	5.7 (5.5−5.9)	10.0 (9.8−10.3)	8.1 (7.7−8.4)	10.8 (10.4−11.1)
Leukemia	C91−95	59	8.5 (8.1−9.0)	13.4 (13.0−13.9)	7.7 (7.3−8.1)	12.7 (12.3−13.1)	9.7 (9.2−10.2)	14.4 (13.9−14.9)
All	C00−97	64	8.8 (8.7−9.0)	9.2 (9.0−9.3)	6.7 (6.6−6.8)	9.8 (9.7−9.9)	11.2 (11.1−11.4)	8.4 (8.3−8.6)

CI. Confidence interval; ICD-10. International classification of diseases 10th revision

### 3.2 LE and YLL varied by the age at diagnosis

**Fig. 1:**
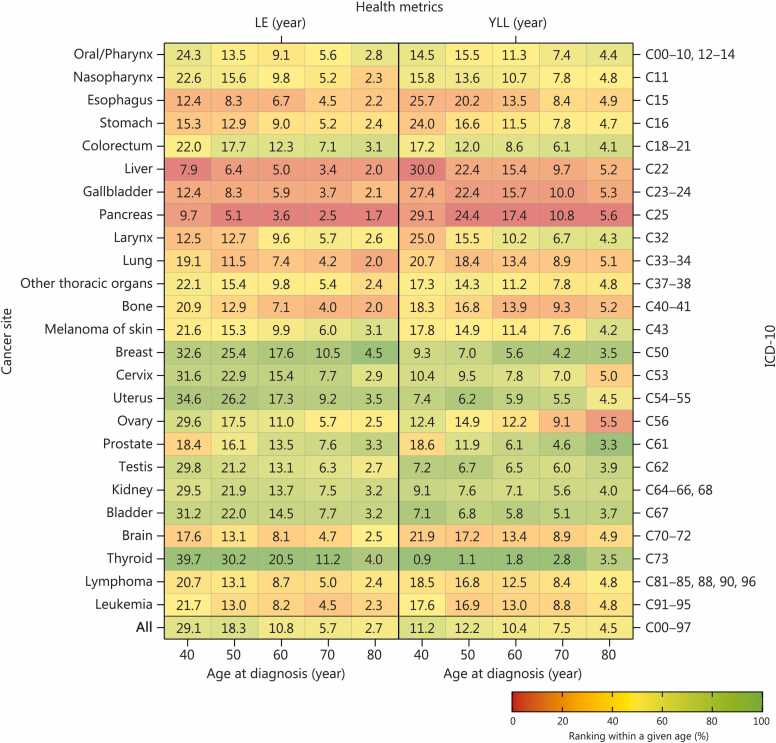
Life expectancy (LE) and years of life lost (YLL) among cancer patients according to age at diagnosis, for both sexes. For a given cancer type, LE and YLL for both sexes were adjusted by the male-to-female ratio of its cancer cases. ICD-10. International Classification of Diseases, 10th revision.

LE and YLL estimates varied by age at cancer diagnosis (Fig. 1). Patients diagnosed at ages 40, 50, 60, 70, and 80 years had LEs of 29.1 years (95% CI 28.6−29.5), 18.3 years (95% CI 18.0−18.6), 10.8 years (95% CI 10.6−10.9), 5.7 years (95% CI 5.6−5.8), and 2.7 years (95% CI 2.6−2.7), respectively, with corresponding YLLs of 11.2 years (95% CI 10.8−11.7), 12.2 years (95% CI 11.9−12.4), 10.4 years (95% CI 10.2−10.5 years), 7.5 years (95% CI 7.4−7.6), and 4.5 years (95% CI 4.5−4.5). Generally, earlier diagnosis was associated with higher LE, primarily reflecting the inherent LE of the general population. However, because cancer-induced mortality reduced LE in cancer patients, younger patients occasionally demonstrated worse survival, resulting in lower LEs for certain cancers compared to older patients. For example, laryngeal cancer patients diagnosed at age 40 had a lower LE than those diagnosed at age 50 years [12.5 (95% CI 10.6−14.5) vs. 12.7 (95% CI 11.7−13.8)]. Regarding YLL, the YLL were generally comparable before age 60 but decreased obviously thereafter, demonstrating the growing impact of competing mortality from other diseases in older populations.

### 3.3 LE and YLL varied by sex

**Fig. 2:**
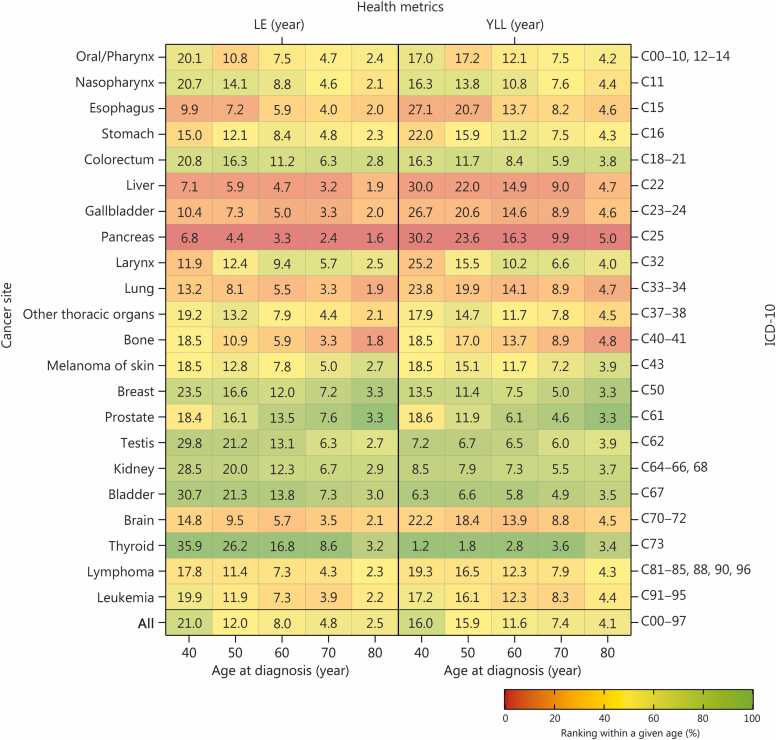
Life expectancy (LE) and years of life lost (YLL) among cancer patients according to age at diagnosis, for males. LE for the general population was estimated for males only. ICD-10. International Classification of Diseases, 10th revision.

**Fig. 3:**
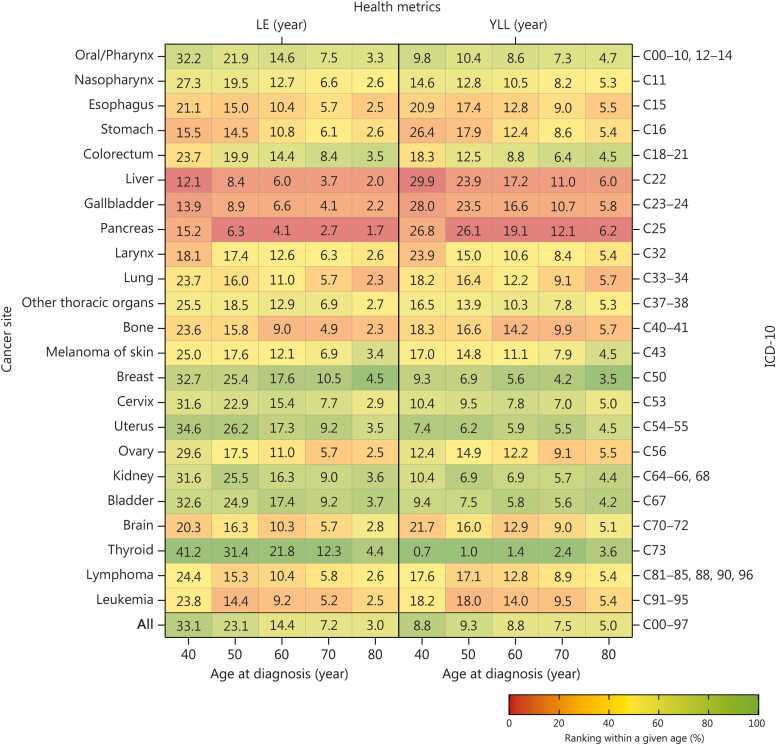
Life expectancy (LE) and years of life lost (YLL) among cancer patients according to age at diagnosis, for females. LE for the general population was estimated for females only. ICD-10. International Classification of Diseases, 10th revision.

LE and YLL estimates also varied by sex. The differences in estimates between male (Fig. 2) and female (Fig. 3) cancer patients represented both inherent LE disparities between general populations by sex and survival differences between male and female cancer patients. For male patients diagnosed at ages 40, 50, 60, 70, and 80 years, LEs were 21.0 years (95% CI 20.6−21.5), 12.0 years (95% CI 11.8−12.3), 8.0 years (95% CI 7.9−8.2), 4.8 years (95% CI 4.7−4.9), and 2.5 years (95% CI 2.4−2.5), respectively, with corresponding YLLs of 16.0 years (95% CI 15.5−16.5), 15.9 years (95% CI 15.7−16.2), 11.6 years (95% CI 11.4−11.7), 7.4 years (95% CI 7.4−7.5), and 4.1 years (95% CI 4.1−4.2). For female patients diagnosed at the same ages, LEs were 33.1 years (95% CI 32.7−33.6), 23.1 years (95% CI 22.8−23.4), 14.4 years (95% CI 14.2−14.6), 7.2 years (95% CI 7.1−7.3), and 3.0 years (95% CI 2.9−3.0), respectively, with corresponding YLLs of 8.8 years (95% CI 8.4−9.3), 9.3 years (95% CI 8.9−9.6), 8.8 years (95% CI 8.6−9.0), 7.5 years (95% CI 7.4−7.6), and 5.0 years (95% CI 5.0−5.1).

### 3.4 LE and YLL varied by attained age

**Fig. 4:**
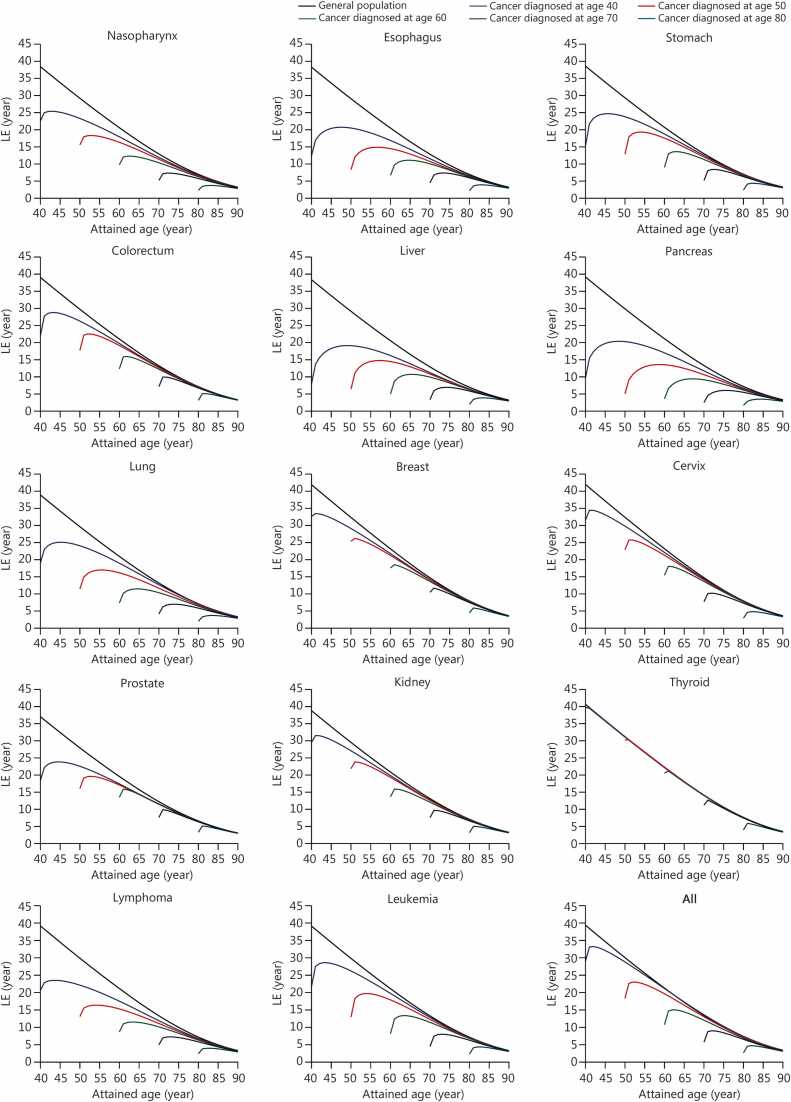
Life expectancy (LE) of general population and cancer patients according to attained age, for both sexes. Only all cancers and the 14 selected major cancer types are shown. Attained age is the age a survivor has reached at a given time. For a given cancer type, general population’s LE was adjusted by the male-to-female ratio of its cancer cases. Since females have a better LE, cancers with a higher proportion of female cases correspond to a higher general population LE. The difference in LE between cancer patients and the general population represents the years of life lost (YLL).

LE and YLL further varied by attained age (Fig. 4**, and Additional file 2:** Fig. S1). The LE of the general population declined gradually with increasing attained age, whereas cancer patients demonstrated a distinct pattern: their LE was initially low in the first few years after diagnosis, then gradually increased, and eventually reproduced the declining trajectory of the general population’s LE. The YLL estimates were highest at the year of cancer diagnosis and declined gradually with attained age, eventually approaching zero.

## 4 Discussion

In this population-based comparative study in China, we estimated LE for 26 cancer types by sex, age at diagnosis, and attained age, quantifying YLL for cancer patients through comparison with a sex- and age-matched general population. In 2021, cancer patients diagnosed at the median age of 64 had a LE of 8.8 years and a YLL of 9.2 years. LE and YLL estimates varied by sex, cancer type, age at diagnosis, and attained age.

Cancer patients, particularly those diagnosed with cancers associated with lower survival rates, experience a substantially higher excess mortality risk. The excess mortality risk in cancer patients arises from multiple aspects, including both acute events, such as vascular coagulation, cardiac failure, displacement, functional impairment or obstruction of vital organs, infections, paraneoplastic syndromes, and therapy-induced toxicity, as well as underlying causes, such as dysfunction of the immune, hematopoietic, and the nervous systems, metabolism and cachexia, and whole-body dysfunction [32]. Cancer patients are also at high risk of dying from non-cancer causes [33], [34]. For instance, those with prostate or breast cancer often have elevated mortality from conditions such as heart disease (either treatment-related or due to general risk factors) or infections (frequently secondary to treatment) [33]. The risk of non-cancer mortality was especially elevated during the first year following cancer diagnosis [33], [34]. Compared to the general population, cancer patients experienced a 10- to 10,000-fold higher relative risk of non-cancer death within the first year after cancer diagnosis [33]. These time-varying excess mortality risks were captured by LE estimates, which may explain the observed variations in LE across attained ages.

Cancer types with a higher average excess mortality risk after diagnosis, i.e., lower 5-year relative survival, generally have lower LE. In China, pancreatic cancer has the lowest age-standardized 5-year relative survival at 8.5%, and consequently the lowest LE at diagnosis (2.6 years) [23]. In contrast, thyroid cancer has the highest 5-year relative survival (92.9%), leading to the highest LE at diagnosis (31.9 years) [23]. For most cancers, a younger age at diagnosis was associated with higher LE, except for laryngeal cancer. This is because older patients typically have lower 5-year relative survival rates compared to younger patients. However, prostate cancer, stomach cancer, colorectal cancer, and laryngeal cancer represented lower 5-year survival rates when diagnosed before age 45 than at age 50. Similarly, for some cancers, younger patients may experience fewer YLLs due to their lower excess mortality risk compared to older patients. For other cancers, however, younger patients may lose more YLLs because they experienced a longer period of excess mortality and were exposed to lower competing mortality risks. For a given cancer type, male patients had lower LE and generally higher YLL than female patients. Potential reasons for sex differences in LE and YLL are likely similar to those underlying differences in cancer survival rates. These include genetic and endocrine factors, treatment received, health behaviors, and tumor characteristics such as histologic subtype and stage at diagnosis [14], [35]. The LE of cancer patients was initially low after diagnosis, then gradually increased, eventually approaching that of the general population. This pattern resulted from the trend in excess mortality risk among cancer patients after diagnosis. Typically, cancer patients experienced the highest excess mortality risk in the initial years after diagnosis. As survival time increased, this excess risk gradually declined among cancer survivors.

Years of YLL among Chinese cancer patients demonstrated some heterogeneity compared to those in other countries or areas. In cancer patients diagnosed at age 55 in the United States in 2010, the YLL for male lung cancer and colorectal cancer were 22.2 and 8.6 years, respectively, while for female lung cancer, colorectal cancer, and breast cancer were 23.2, 8.9, and 3.8 years [5]. The YLL for female breast cancer in the United States was slightly lower than that in China, whereas the estimates for lung cancer were higher, and colorectal cancer was comparable. In England between 1998 and 2013, the YLL for male lung cancer, stomach cancer, colorectal cancer, and prostate cancer, with an average diagnosis age of about 71 years, was approximately 12.0, 10.5, 6.5, and 2.5 years, respectively [10]. For female breast cancer, with an average diagnosis age of about 63 years, the loss was around 5.5 years [10]. These findings suggested that among older cancer patients, China’s YLL for male lung cancer, stomach cancer, and colorectal cancer was lower than England, while the estimates for prostate cancer were higher, and female breast cancer was comparable. In Australia between 1990 and 2007, the YLL for cancer patients diagnosed at ages 40 and 80 was 11.2 and 3.9 years, respectively [19]. These estimates were similar to our estimates for Chinese patients.

These disparities in YLL across countries or regions may be attributed to the combined effects of treatment factors and patient characteristics. With respect to treatment-related factors, the cancer patients included in YLL estimations vary by country (or region) in terms of their diagnosis years, and there are also disparities in the health literacy, healthy behaviors, socioeconomic status, and accessibility of standardized treatment [10], [11], [12]. While we only controlled for age and sex when comparing YLL across different countries (or regions), numerous patient-specific characteristics can influence YLL, including anatomical subsite, cancer stage, tumor grade, biomarkers, and histologic subtype [13], [14]. Regarding LE, variations among countries were more noticeable because of substantial differences in the LE of the general population. For example, Japan, which has the world’s highest LE, reported that patients diagnosed with early gastric cancer at age 40 had a LE as high as 45 years [36].

Estimating LE for cancers require the construction of life tables specific to these patients [5], [17]. In contrast, YLL due to cancer can be calculated indirectly through counterfactual assumptions by eliminating cancer mortality rates from the LE of the general population [21], [37]. Nevertheless, this approach has several limitations. Firstly, it does not account for the substantial non-cancer mortality risks among cancer patients [33], [34]. Secondly, the indirect method does not take into account how the excess mortality risk of cancer patients changes with age [17], [33]. Thirdly, estimating the potential YLL per cancer case is challenging because the calculation does not incorporate cancer incidence data [21], [38]. In China, many studies utilize this indirect method to estimate YLL [21], [39], [40]. Generally, these reports relied on health metrics like total YLL, the rate of YLL, and the average YLL per cancer death. These metrics differ in meaning from the life years lost directly estimated in our study, which was calculated based on the relative survival or excess mortality risk of cancer patients (i.e., by explicitly excluding the impact of cancer mortality from the general population’s overall mortality risk) YLL [5], [17].

Our study should be interpreted with consideration of its limitations. Firstly, the LE were estimated by the period life table method, which was based on the cross-sectional mortality data in 2021. Clearly, using cross-sectional death rates transformed into probabilities of dying, under the hypothetical scenario that the prevailing age-specific death rates remain constant in the future, may differ from the actual longitudinal mortality risk of the population [41]. The ideal approach for LE calculation would be cohort-based methods; however, long-term follow-up until all individuals have died is impractical. This is why period LE is more frequently used than cohort LE [41]. Secondly, there was uncertainty in the long-term excess hazard functions of cancer patients. We only included variations in the 5-year relative survival rates of Chinese cancer patients, while longer-term variances need validation through future long-term follow-up data from Chinese cancer cohorts. Thirdly, we did not consider the variations in LE and YLL among different regions and socioeconomic statuses [10], [42]. Finally, we only reported the LE and YLL for the 26 most common cancer types, excluding rare cancers or the LE estimates for childhood cancers [3], [18]. In the future, as data continues to build, it will be essential to assess the disparities within these minority groups.

## 5 Conclusions

Cancer patients in China experienced a substantial loss of life years after diagnosis, with LE varying by sex, cancer type, age at diagnosis, and attained age. Health metrics related to cancer patient LE and life loss were important supplements to cancer incidence, mortality, and relative survival rates. These metrics may be utilized by cancer patients, healthcare providers, and health policymakers to inform their decisions regarding cancer care.

## Abbreviations

CI: Confidence interval

LE: Life expectancy

YLL: Years of life lost

## Ethics approval and consent to participate

This study was conducted at the aggregate level and did not involve any personal or medical information from identifiable individuals. It was approved by the Ethics Committee of National Cancer Center/Cancer Hospital, Chinese Academy of Medical Sciences, and Peking Union Medical College (22/489-3691).

## Consent for publication

Not applicable.

## Authors’ contributions

CFX obtained funding for the study. WQC designed the study. CFX and JHZ collected the data. JY cleaned and verified data. CFX analyzed the data. WQC and JY supervised the analysis and generation of the results. CFX drafted and finalized the manuscript. WQC, JY, JHZ, and YJX interpreted the results and critically revised the manuscript. All authors have read and approved the final manuscript.

## Funding

This study was supported by the National Key R&D Program of China (2022YFC3600805).

## Competing interests

The authors declare that they have no conflict of interests.

## Footnotes

Appendix A Supplementary data associated with this article can be found in the online version at doi:10.1016/j.mmr.2026.100045.
