# Supplementary material for: Life expectancy of cancer patients in China
Source: Mil Med Res. 2026 Jun 15;13(1):100045. doi: 10.1016/j.mmr.2026.100045 (PMC13285705; doi:10.1016/j.mmr.2026.100045)
Supplement: Supplementary file 2 — Supplementary material [file mmc2.pdf]

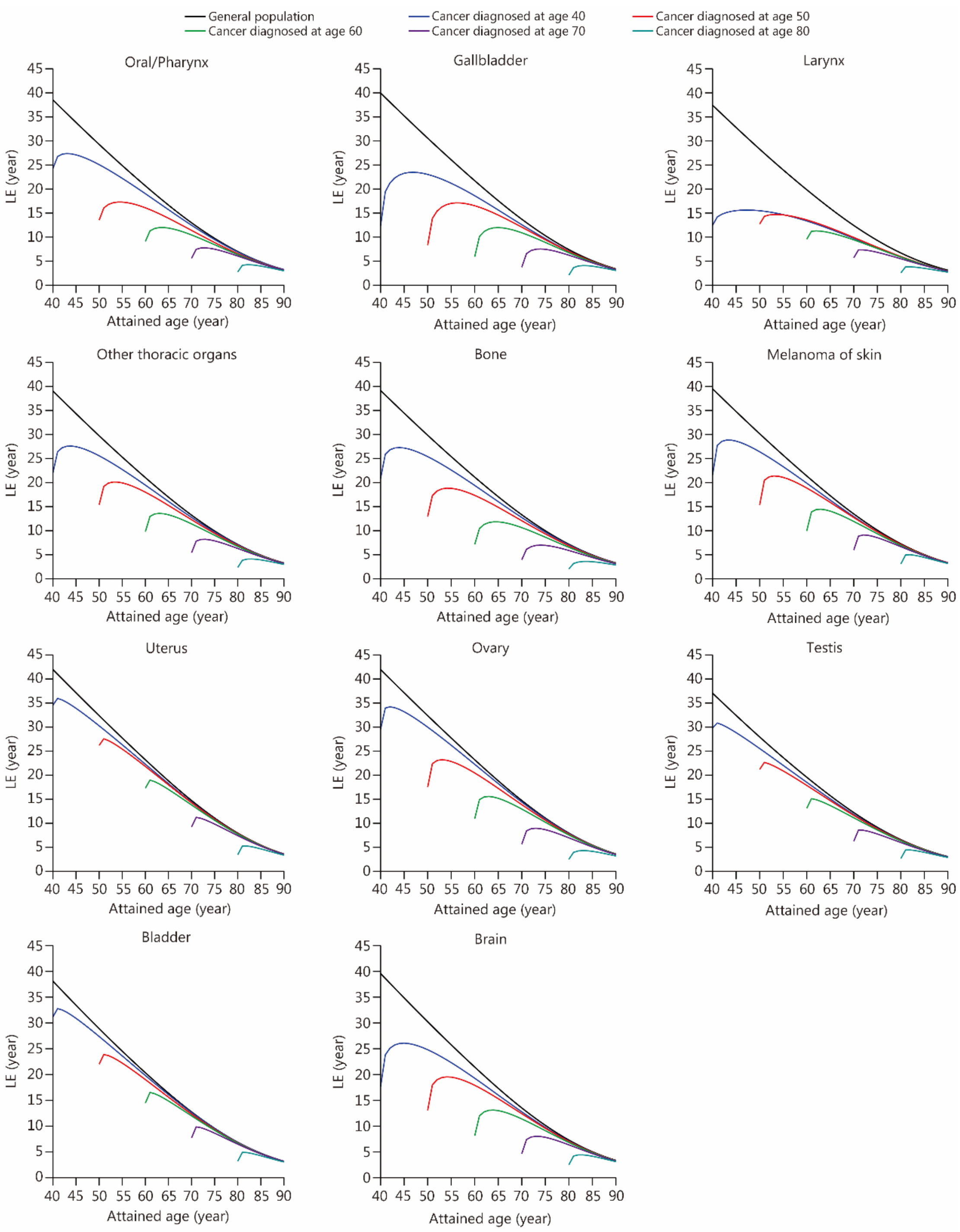

Fig. S1 Life expectancy (LE) of general population and cancer patients according to attained age, for remaining 11 cancer types. Attained age is the age a survivor has reached at a given time. For a given cancer type, general population's LE was adjusted by the male-to-female ratio of its cancer cases. Since females have a better LE, cancers with a higher proportion of female cases correspond to a higher general population LE. The difference in LE between cancer patients and the general population represents the years of life lost (YLL)
